# Reactive Sulfur Species in Biology and Medicine

**DOI:** 10.3390/antiox12091759

**Published:** 2023-09-13

**Authors:** Vinayak S. Khodade, John P. Toscano

**Affiliations:** Department of Chemistry, Johns Hopkins University, Baltimore, MD 21218, USA

Hydrogen sulfide (H_2_S) has emerged as a third small-molecule bioactive signaling agent, along with nitric oxide (NO) and carbon monoxide (CO). It regulates diverse physiological processes such as cell differentiation, development, and immune responses. H_2_S is produced mainly by cystathionine-β-synthase (CBS), cystathionine-γ-lyase (CSE), and 3-mercapto-sulfurtransferase (3-MST) in mammals. Despite being highly toxic at elevated concentrations, emerging evidence highlights a distinct cytoprotective role of endogenously produced H_2_S, and intensive studies have focused on its potential as a therapeutic agent for cardiovascular, inflammatory, infectious, and neuropathological diseases. Furthermore, emerging evidence shows that many biological effects initially ascribed to H_2_S may instead be due to other reactive sulfur species (RSS), including hydropersulfides (RSSH) and other higher-order polysulfur species (RSS_n_H, RSS_n_R, HS_n_H, n > 1). Importantly, these RSS are highly prevalent in biological systems. RSS have intriguing biochemical properties, such as the ability to modify protein cysteine residues and efficiently scavenge reactive oxygen species and electrophiles. Moreover, RSS donors have begun to demonstrate therapeutic potential in treating a range of maladies, including cancer, neurological disorders, and cardiovascular disease. Nonetheless, the chemistry of RSS is complicated due to their reactive nature, and their accurate measurement in biological systems remains a challenge. This Special Issue focuses on studies that highlight recent advances in H_2_S/RSS chemical biology, including six original research articles and five reviews aimed at exploring the therapeutic applications of H_2_S/RSS donors, understanding the molecular mechanisms and physiological roles of RSS, and methods that measure the concentration and distribution of RSS in biological systems.

Paul and Pieper summarize the latest discoveries regarding the neuroprotective function of H_2_S in Alzheimer’s disease (AD) and traumatic brain injury (TBI) [1]. Initially, they delve into H_2_S biosynthesis in the brain, followed by an exploration of its role in these major neuropsychiatric conditions characterized by cognitive impairment. The authors review the existing literature that underscores the significance of adequate H_2_S signaling in modulating neuronal health and reveal how deficiencies in this signaling molecule contribute to the pathology of AD and TBI. They also highlight the mechanism of the underlying physiological effects of H_2_S through the persulfidation of cysteine residues on target proteins. The authors underscore the potential of H_2_S donors as therapeutic agents in alleviating symptoms associated with AD, TBI, and analogous neurodegenerative disorders characterized by compromised H_2_S signaling pathways.

H_2_S also plays a pivotal role in the cardiovascular system, exerting profound influence over vasodilation and blood pressure regulation. Lukesh and Hu present a comprehensive review that delves into H_2_S donors with promising cardioprotective effects against myocardial ischemia-reperfusion (MI/R) injury and chemotherapy-induced cardiotoxicity, a frequent and often lethal complication of chemotherapy [2]. They provide a systematic categorization of donors based on mechanisms triggering H_2_S release, encompassing hydrolysis, pH, thiol, enzyme, and ROS. A detailed explanation of the H_2_S-releasing mechanism for each donor is provided, along with an overview of their therapeutic effects across various models of MI/R injury. The potential benefits of H_2_S and H_2_S-conjugated codrugs in mitigating anthracycline-induced cardiotoxicity are also discussed.

Recent research has indicated the potential benefits of H_2_S in alleviating hypertension. In this context, Hsu and colleagues present their study that investigates the protective effects of sodium thiosulfate (STS) in a rat model of hypertension induced by maternal chronic kidney disease (CKD) [3]. Thiosulfate, a member of the sulfane sulfur family, is known to undergo enzymatic reduction to generate H_2_S. The researchers induced CKD in rats by exposing them to a diet enriched with 0.5% adenine for three weeks prior to mating. Subsequently, pregnant rats received sodium thiosulfate (2 g/kg/day) during both gestation and lactation. The study demonstrated that administering STS effectively counteracted the development of hypertension in the offspring. Employing HPLC–mass spectrometry, the investigators observed significant increases in the plasma H_2_S concentrations and renal tissue levels of the 3MST protein in the CKD offspring. The authors suggest that the positive effects of STS treatment can be attributed, at least in part, to enhancements in the signaling pathways of H_2_S and NO. Furthermore, the researchers delved into the impact of STS on the composition of gut microbiota. A particularly intriguing finding was that maternal CKD led to a reduction in the population of the *Enterococcus* genus, coupled with an elevation in the abundance of *Erysipelatoclostridium* and *Dorea* genera. Conversely, administering maternal rats with STS resulted in higher proportions of the *Dorea*, *Streptococcus*, and *Anaerotruncus* genera. Importantly, the favorable impact of STS on offspring hypertension correlated with an increase in beneficial microbial species. Thus, this study underscores the potential efficacy of early-life STS intervention in mitigating hypertension among offspring born to mothers with CKD.

H_2_S-releasing compounds have also emerged as potential therapeutics for various types of cancers. Ianaro, Ercolano, and co-workers examine the effects of erucin (ERU), a compound derived from the dietary source, on human melanoma cell lines [4]. ERU was found to exert a significant inhibitory effect on human melanoma cell lines. Notably, ERU was shown to induce apoptosis in A375 melanoma cells. Expanding on ERU’s proapoptotic attributes, the researchers delved into its effect on cadherins and transcription factors central to the epithelial-to-mesenchymal transition (EMT) process, a critical event in cancer progression. They demonstrated that ERU significantly enhances the expression of the epithelial protein E-CAD while reducing the expression of the mesenchymal protein N-CAD. Beyond its effects on apoptosis and cell cycle regulation, ERU exhibited remarkable inhibition of A375 melanoma cell migration, invasiveness, and clonogenic potential. Intriguingly, ERU also demonstrated the ability to diminish melanin levels and downregulate genes associated with melanogenesis, hinting at its potential to interfere with melanoma-specific pathways. An intriguing facet of ERU’s mechanism was its capacity to attenuate the generation of intracellular ROS in A375 cells.

Beyond its presence in mammals, H_2_S is also produced by bacteria. Recent studies have demonstrated its pivotal role in bacterial physiology, particularly in bacterial responses toward host-induced stressors like ROS and antibiotics during infections. Recent studies, however, suggest that the physiological benefits of H_2_S are likely attributed to its downstream sulfur derivatives, i.e., RSS. Brito, Giedroc, and co-workers present their investigation of H_2_S/RSS homeostasis in the microbial pathogen *Enterococcus faecalis*, probing the pathophysiological response of elevated H_2_S levels [5]. Employing proteomic analysis, they demonstrated a notable increase in the cellular abundance of enzymes reliant on coenzyme A (CoA) and acyl-CoA. They also examined protein persulfidation, revealing that approximately 13% of the proteome undergoes persulfidation and is significantly augmented by exogenous sulfide introduction. Prominent among the proteins persulfidated were CstR, a sensor for RSS, and CoAPR, an enzyme intricately associated with the reduction of coenzyme A persulfide (CoASSH). Furthermore, they demonstrated the impact of exogenous sulfide on the speciation of fatty acid composition and cellular acetyl-CoA concentrations. Collectively, these insights augment our understanding of the intricate interplay encompassing H_2_S/RSS signaling, protein persulfidation, and the metabolic pathways governed by coenzyme A and acyl-CoA in *Enterococcus faecalis*.

The molecular mechanisms underlying the physiological effects of H_2_S are complex and remain an active area of research. Markel and colleagues present a review that delves into the molecular and cellular mechanisms of H_2_S within mammalian systems [6]. In their exploration of potential direct targets, the authors focus on H_2_S interactions with reactive oxygen/nitrogen species, which is critical in redox signaling. Furthermore, H_2_S interaction with hemeproteins and its role in modulating metal ions containing complexes are highlighted. The post-translational modification of cysteine residue in proteins via oxidized derivative of H_2_S, i.e., persulfidation, is emphasized. The H_2_S-mediated significant impact on cell structure and many cellular functions, such as tight junctions, autophagy, apoptosis, vesicle trafficking, cell signaling, epigenetics, and inflammasomes is discussed. Importantly, the authors also indicate that several biological effects initially ascribed to H_2_S might instead be attributed to other RSS. In recent years, RSS has remained an active area of research with a focus on their biosynthetic pathways and physiological functions.

Recently, Akaike and colleagues have revealed that cysteinyl-tRNA synthetase (CARS) functions as a cysteine hydropersulfide synthase (CPERS), which is primarily responsible for generating RSSH in biological systems. However, some researchers have proposed that H_2_S-producing enzymes 3-MST, CBS, and CSE also contribute to RSSH production. To determine hydropersulfide biosynthesis, Tsutsui, Akaike, and co-workers conducted research to elucidate the relative contributions of these enzymes to cysteine hydropersulfide biosynthesis [7]. They initiated their investigation by creating 3-MST knockout mice, which surprisingly exhibited no statistically significant differences in RSSH production when compared to wild-type mice. Then, they generated triple-knockout mice lacking the CBS, CSE, and 3-MST genes by crossbreeding them with single-knockout mice of each of these enzymes. Using LC–MS/MS analysis, they quantified levels of CysSSH and GSSH in various tissues and plasma from both the triple-knockout and control mice. Remarkably, the absence of CBS, CSE, and 3-MST genes did not appear to hinder RSSH synthesis. The researchers also turned their attention to CARS2-deficient heterozygous mice (*Cars2^+/−^*) mice and found significantly reduced levels of CysSSH, GSSH, and related compounds in liver and lung tissues of these mice compared to their wild-type counterparts, pointing to the reliance on CARS2 for the synthesis of RSSH and sulfide derivatives in mouse tissues. Western blot assays conducted on tissues from *Cars2^+/−^* mice indicated unchanged protein expression levels of 3-MST, CBS, and CSE between wild-type and CARS2-deficient mice, suggesting the absence of compensatory upregulation in the expression of these canonical enzymes. Thus, this study highlights the critical role of CARS as the primary enzyme responsible for biosynthesizing RSSH. The findings provide substantial evidence regarding the relative involvement of various enzymes in RSSH production, clarifying the complex landscape of sulfur metabolism in biological systems.

In the last decade, RSS donors have been tested for their therapeutic potential across a spectrum of disorders, including cancer, neurological disorders, and cardiovascular disease. Ichinose and co-workers present a study examining the potential neuroprotective effects of glutathione trisulfide (GSSSG) within a murine model of paclitaxel-induced peripheral neuropathy (PIPN) [8]. These researchers showed that oral administration of GSSSG at 50 mg/kg/day over 28 days effectively mitigates the mechanical allodynia induced by PIPN. Through liquid chromatography coupled with tandem mass spectrometry analysis, the study demonstrated the presence of ^34^S-labeled GSSSG within the lumbar dorsal root ganglia (DRG) and lumbar spinal cord for two hours following oral administration. Additionally, the researchers showcased several pivotal outcomes in paclitaxel-treated mice following GSSSG administration. These included the upregulation of antioxidant gene expression in the lumbar DRG, the preservation of unmyelinated axons, and the attenuation of sciatic nerve mitochondria degeneration. Moreover, GSSSG effectively counteracted paclitaxel-induced superoxide generation, mitigated the loss of axonal mitochondria, and attenuated axonal degeneration in primary neurons derived from both the cortex and DRG. These collective findings strongly suggest that the oral administration of GSSSG effectively counters PIPN by safeguarding peripheral sensory nerve axons and maintaining the structural integrity of mitochondria. 

Within the domain of redox biology, biological sulfane sulfur (S^0^) species, including RSSH, RSS_n_SR, H_2_S_n_, n ≥ 2, and protein-bound elemental sulfur (S_8_), have emerged as pivotal RSS. Despite their important roles in redox signaling, the exploration of sulfane sulfur species remains difficult, given their inherent instability and low concentrations in biological systems. Xian and co-workers present a review article that delves into organelle-targeted fluorescent probes for sulfane sulfur species [9]. The scope of the review encompasses a variety of probes designed for distinct organelles, including mitochondria, endoplasmic reticulum, and lysosomes. The strategies employed to direct these probes to specific cellular locales are thoroughly examined. An overview of organelle-targeted sulfane sulfur probes is presented, encompassing their underlying mechanisms, capacity for colocalization, sensitivities, and efficacy in detecting endogenous and exogenous sulfane sulfur under physiological circumstances. The authors highlight a predominant focus on mitochondria among the organelle-targeted probes, with relatively limited attention directed toward lysosomes and the endoplasmic reticulum. The authors further identify a significant gap in the availability of sulfane sulfur probes for additional organelles, such as the Golgi apparatus and nucleus, thereby proposing directions for future research. 

Beyond the modification of cysteine residues on proteins through interactions with sulfane sulfur species, ROS also mediate the oxidative post-translational modifications of cysteines that are pivotal in cell signaling. Notably, ROS-mediated cell signaling is particularly prominent within mitochondria, given that ROS constitute natural byproducts of cellular respiration. While various sites of cysteine oxidation on mitochondrial proteins have been identified, a notable interest in identifying uncharacterized redox-sensitive cysteines persists. In a contribution by Weerapana and colleagues, an approach combining mitochondrial enrichment with redox proteomic methodologies was employed to identify the cysteines sensitive to redox alterations by ROS [10]. They utilized an isotopic tandem orthogonal proteolysis-activity-based protein profiling (isoTOP-ABPP) platform for the indirect identification of oxidation sites by tracking the loss of cysteine reactivity. Additionally, the oxidative isotopically coded affinity tag (OxICAT) platform was employed to monitor reversible cysteine oxidation through differential labeling. Within the isoTOP-ABPP framework, mitochondrial cysteines were systematically evaluated for their responsiveness to hydrogen peroxide exposure. The outcomes revealed a set of approximately 700 mitochondrial cysteines exhibiting varying degrees of susceptibility to peroxide-triggered oxidation. These cysteines were effectively classified into categories of low, moderate, and high sensitivity, revealing distinct patterns of reactivity. Furthermore, the study explored the spatial distribution of redox-sensitive cysteines within crucial mitochondrial pathways. Particularly, the tricarboxylic acid (TCA) cycle and proteins involved in mitochondrial transcription and translation were noted for harboring noteworthy concentrations of highly redox sensitive cysteines. 

Emerging evidence suggests that reactive species generated from the metabolism of oxygen, NO, and H_2_S have the potential to interact with each other. This interaction, in turn, has the capacity to influence shared biological targets. These complex interplays are termed the “reactive species interactome”. Cortese-Krott presents a review article that examines the reactive species interactome, specifically focusing on its relevance to the intricate landscape of redox biology in red blood cells (RBCs) [11]. The review investigates multiple facets, including the generation and detoxification of reactive species, their interactions, and their ultimate molecular targets. It is also noted that future research endeavors should be directed towards unraveling how modulation of the reactive species interactome intricately regulates the biology, physiology, and systemic repercussions of RBCs. 

In summary, this Special Issue compiles original research articles as well as review articles that collectively illuminate the rich significance of H_2_S and RSS within biological systems. These reports serve not only as a testament to the current state of research, but also as a catalyst for the further exploration of important, unanswered questions. 
